# Life on a Mesoarchean marine shelf – insights from the world’s oldest known granular iron formation

**DOI:** 10.1038/s41598-020-66805-0

**Published:** 2020-06-29

**Authors:** Albertus J. B. Smith, Nicolas J. Beukes, Jens Gutzmer, Clark M. Johnson, Andrew D. Czaja, Noah Nhleko, Frikkie de Beer, Jakobus W. Hoffman, Stanley M. Awramik

**Affiliations:** 1 Paleoproterozoic Mineralization Research Group, Department of Geology, University of Johannesburg, Johannesburg, South Africa; 2 Department of Science and Technology-National Research Foundation Centre of Excellence for Integrated Mineral and Energy Resource Analysis, University of Johannesburg, Johannesburg, South Africa; 3 Helmholtz Zentrum Dresden-Rossendorf, Helmholtz Institute Freiberg for Resource Technology, Freiberg, Germany; 4 Department of Mineralogy, TU Bergakademie Freiberg, Freiberg, Germany; 5 Department of Geoscience, University of Wisconsin, Madison, WI USA; 6 Department of Geology, University of Cincinnati, Cincinnati, OH USA; 7 Geological Survey and Mines Department, Mbabane, Swaziland; 8 Radiation Science, South African Nuclear Energy Corporation SOC Ltd. (Necsa), Pelindaba Industrial Site, Gauteng, South Africa; 9 Senior Research Associate, Department of Anthropology & Development Studies, University of Johannesburg, Johannesburg, South Africa; 10 Department of Earth Sciences, University of California, Santa Barbara, CA USA

**Keywords:** Palaeontology, Sedimentology

## Abstract

The Nconga Formation of the Mesoarchean (~2.96–2.84 Ga) Mozaan Group of the Pongola Supergroup of southern Africa contains the world’s oldest known granular iron formation. Three dimensional reconstructions of the granules using micro-focus X-ray computed tomography reveal that these granules are microstromatolites coated by magnetite and calcite, and can therefore be classified as oncoids. The reconstructions also show damage to the granule coatings caused by sedimentary transport during formation of the granules and eventual deposition as density currents. The detailed, three dimensional morphology of the granules in conjunction with previously published geochemical and isotope data indicate a biogenic origin for iron precipitation around chert granules on the shallow shelf of one of the oldest supracratonic environments on Earth almost three billion years ago. It broadens our understanding of biologically-mediated iron precipitation during the Archean by illustrating that it took place on the shallow marine shelf coevally with deeper water, below-wave base iron precipitation in micritic iron formations.

## Introduction

Iron formation (IF, a chemical sedimentary rock containing at least 15 wt% iron^1,2^) has been at the centre of debate with regards to early life on Earth^3,4^ with numerous authors having suggested bacterially mediated deposition of iron-rich mineral precursors^5–11^. Bacterially mediated oxidation of ferrous iron to ferric iron serves as a viable mechanism for iron oxidation in the oxygen-poor surface environments of the Earth prior to the approximately 2.45–2.32 Ga Great Oxidation Event (GOE)^12,13^. It also serves to explain certain mineral and geochemical features of IF^2,10,14^. Granular iron formation (GIF) is a textural subtype of IF where chemically precipitated precursor sediment has been reworked and deposited as endoclastic sands^2^, and mostly occur between approximately 2.4 and 1.9 Ga^14^. GIFs are particularly important to understand as they potentially record iron redox processes in the shallowest parts of the oceans, as compared to IFs in general, most of which were deposited in deep water. However, biological mediation of IF deposition is still debated due to a lack of direct (i.e. textural) evidence for biological activity in older (>2 Ga) occurrences, with some authors even preferring abiological models for IF deposition^15,16^. This contribution presents the first three-dimensional textural evidence, acquired using micro-focus X-ray computed tomography (µXCT), which provides strong evidence that biological activity played an important role in the deposition of IF. Our evidence comes from the exceptionally well preserved - and oldest known - GIF of the Nconga Formation in the Mesoarchean Mozaan Group of the Pongola Supergroup of southern Africa.

**Figure 1:**
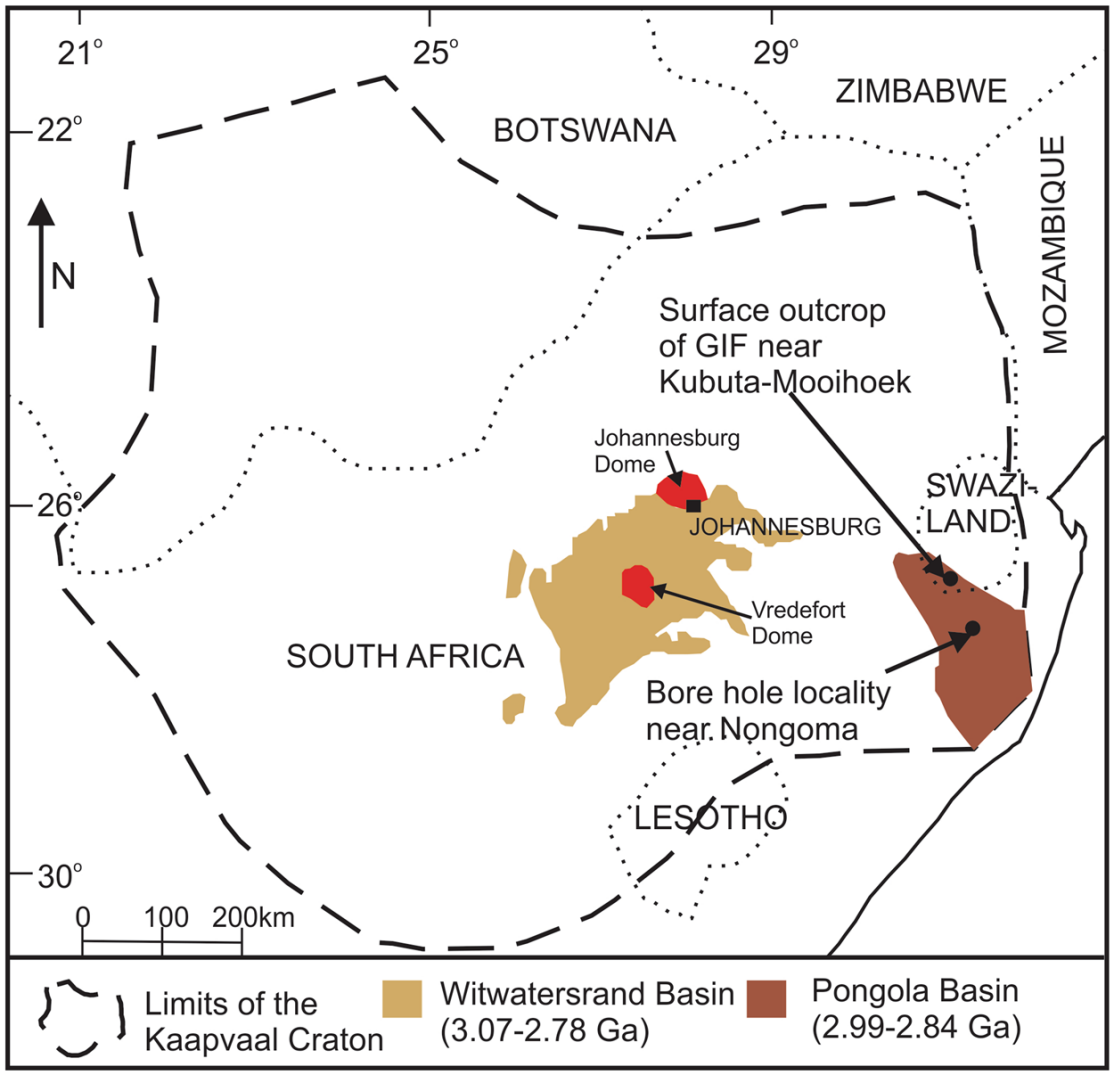
Location of the Pongola Supergroup as well as the studied outcrop and drill core localities on the Kaapvaal Craton of southern Africa. The figure is adapted from^25^. The figure was created using CorelDRAW 2017(www.coreldraw.com).

The Mozaan Group of the Pongola Supergroup (Fig. 1) is one of the oldest supracratonic successions on Earth with a depositional age of approximately 2.96 to 2.84 Ga^17,18^, and, together with the Witwatersrand Supergroup, comprise the oldest well-exposed marine basin known^3,10^. The Mozaan Group is also known to host microbial mat structures in some of its sandstone units^19^. The Mozaan Group contains ten iron-rich sedimentary beds^3^, of which the basal IF in the Vlakhoek Member has been the subject of several recent studies about pre-GOE oxygen oases^20–24^. Less studied, however, is the Nconga Formation, which occurs towards the top of the Mozaan Group. The Nconga Formation contains an iron-rich unit that includes banded IF, massive IF and, importantly, stacked interbeds of GIF; this is the oldest known occurrence of GIF^25^. The granules in the GIF are 0.5 to 3 mm in diameter (Supplementary Figs. 1A and B) and comprise microcrystalline quartz (chert) with minor calcite cores surrounded by magnetite and calcite rims and hosted by a matrix of chert (Supplementary Fig. 1C and D) and minor minnesotaite^25^. The GIF beds are upward fining and capped by iron-rich mudstone (Supplementary Fig. 1A and E; Supplementary Video 1 and 2). Calcite shows depleted δ^13^C_PDB_ values (−15.7 to −11.6 ^0^/_00_), suggestive of diagenetic calcite formation from organic carbon oxidation, and the magnetite is marked by heavy δ^56^Fe values (0.39 to 0.45 ^0^/_00_), indicating partial oxidation of aqueous Fe^2+^ as the main mechanism of iron precipitation^25^. The only iron oxide present in the drill core samples is magnetite, whereas surface samples have been oxidized to hematite^25^. The drill core and outcrop samples show minimal deformation, no evidence of hydrothermal overprint and the metamorphic grade was no higher than lower greenschist facies^25^.

The granule rims are irregular, with multiple magnetite interlayers appearing to mark microdomal structures (Supplementary Fig. 1B–D). The complex structure of the granule rims in the Nconga Formation GIF makes thin section petrography by optical and scanning electron microscope difficult due to the unavoidable bias introduced by two dimensional assessments of three dimensional features (Supplementary Video 3 and 4). The large difference in density between the magnetite in the granule rims and the quartz in the granule cores and matrix, however, presented a unique opportunity to conduct µXCT studies on the Nconga Formation GIF in order to reveal external morphology and textural details.

## Results and granule morphology

**Figure 2:**
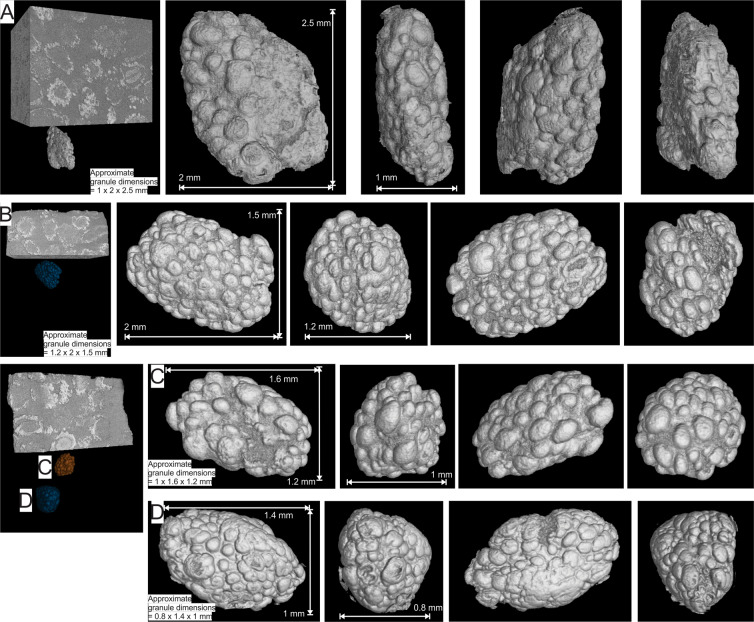
False colour three dimensional reconstructions of µXCT scans of four magnetite coated granules (**A** to **D**) from drill core samples of the Nconga Formation GIF, illustrating, from left to right, the location and orientation of the granules in the sample blocks, and an anti-clockwise rotation (when viewed from top) through each grain. See the following supplementary videos for full rotational animations of the core samples and extracted granules: 5 and 6 for granule A; 7 and 8 for granule B; 9 and 10 for granule C; 9 and 11 for granule D. The images were created using VGStudio Max version 3.2 (https://www.volumegraphics.com/en/products/vgstudio-max.html) and the final figure was compiled using CorelDRAW 2017 (www.coreldraw.com).

The results focus on the false colour 3D µXCT reconstructions of four granules, hereafter referred to as granules A, B, C and D (Fig. 2; Supplementary Videos 5 to 11) from two drill core samples. Supplementary Figures 1A and B are representations of the true colour of the studied samples and the granules, which have black magnetite rims. Granules A and B come from one sample (Fig. 2A and B) and granules C and D from the other sample (Fig. 2C and D). It is important to note that the granules have been imaged in false colour, with denser minerals brighter grey than less dense minerals. So although the outer coating, which comprises magnetite, has been imaged bright grey, the true coating colour is black to dark grey. The granules have the shape of tri-axial ellipsoids, with the three perpendicular axes having different lengths. The granules appear to show no preferred orientation, with the longest axes oriented either vertically (Fig. 2A) or close to horizontally (Fig. 2B to D) with respect to the bedding surfaces. All studied granules have approximately similar sizes and exterior appearances.

**Figure 3:**
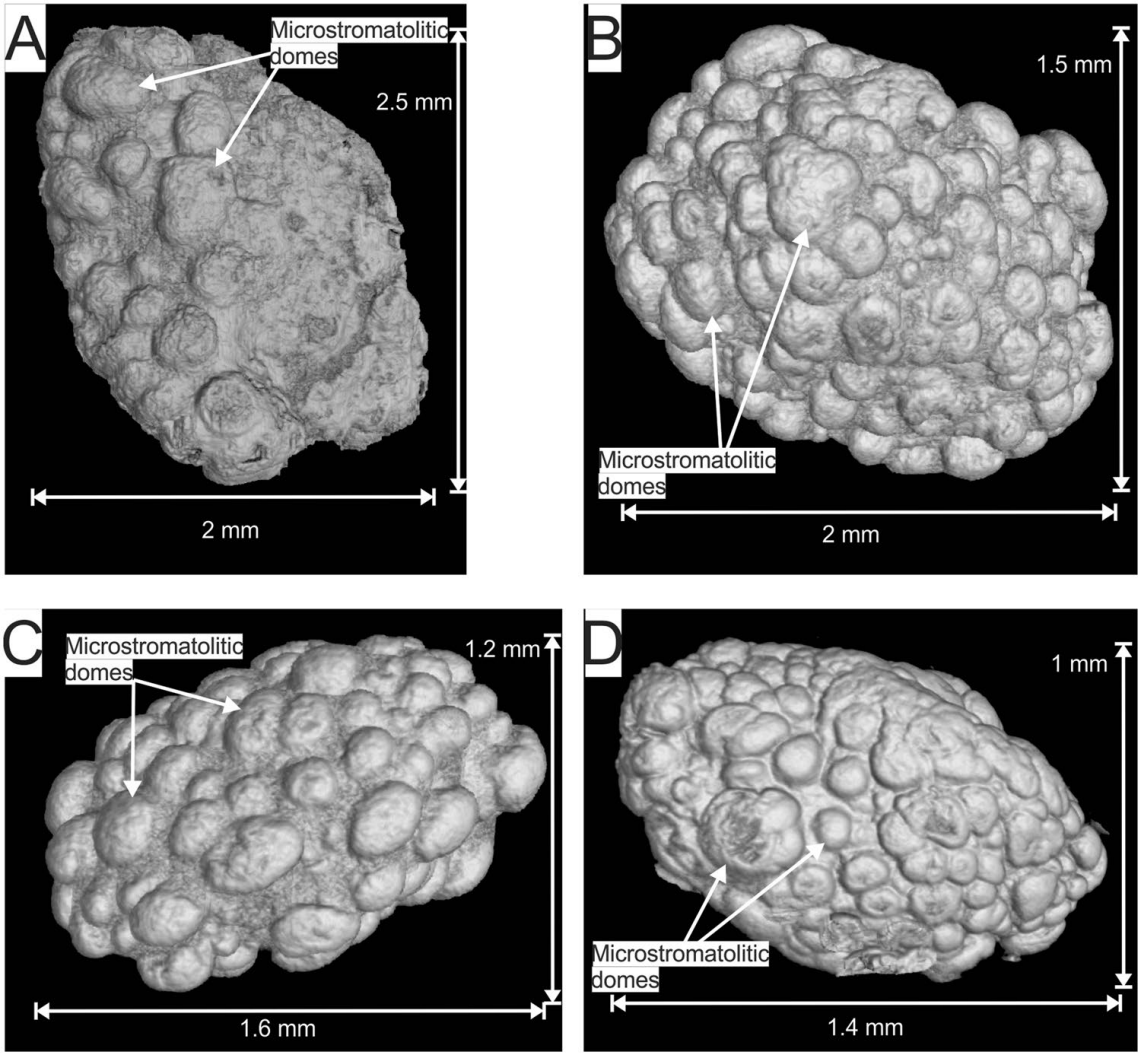
False colour three dimensional reconstructions of µXCT scans of the same four magnetite coated granules from Fig. 2 from drill core samples of the Nconga Formation GIF illustrating the multiple domical structures surrounding, or coating, the outer surface of all the granules. See the following supplementary videos for full rotational animations of the extracted granules: 6 for granule A; 8 for granule B; 10 for granule C; 11 for granule D. The images were created using VGStudio Max version 3.2 (https://www.volumegraphics.com/en/products/vgstudio-max.html) and the final figure was compiled in CorelDRAW 2017 (www.coreldraw.com).

The most prominent feature observed in the 3D µXCT reconstructions, are the multiple domical structures surrounding, or coating, the outer surface of all the Nconga Formation GIF granules, giving them a knobby appearance (Fig. 3; Supplementary Videos 6, 8, 10 and 11). The structures have an outer coating of magnetite with inner laminae comprising calcite and magnetite^25^ (Supplementary Fig. 1). In plan view, the majority of these granule surface structures are circular with a subordinate amount being ellipsoidal (e.g. Fig. 3A and C), with diameters ranging from approximately 30 to 400 μm and with the majority falling in the range of approximately 100 to 300 μm. Although some of the surface structures show partial coalescence (e.g. Fig. 3B), they are mostly separate and occur directly on the outer granule surface and not on top of each other.

**Figure 4:**
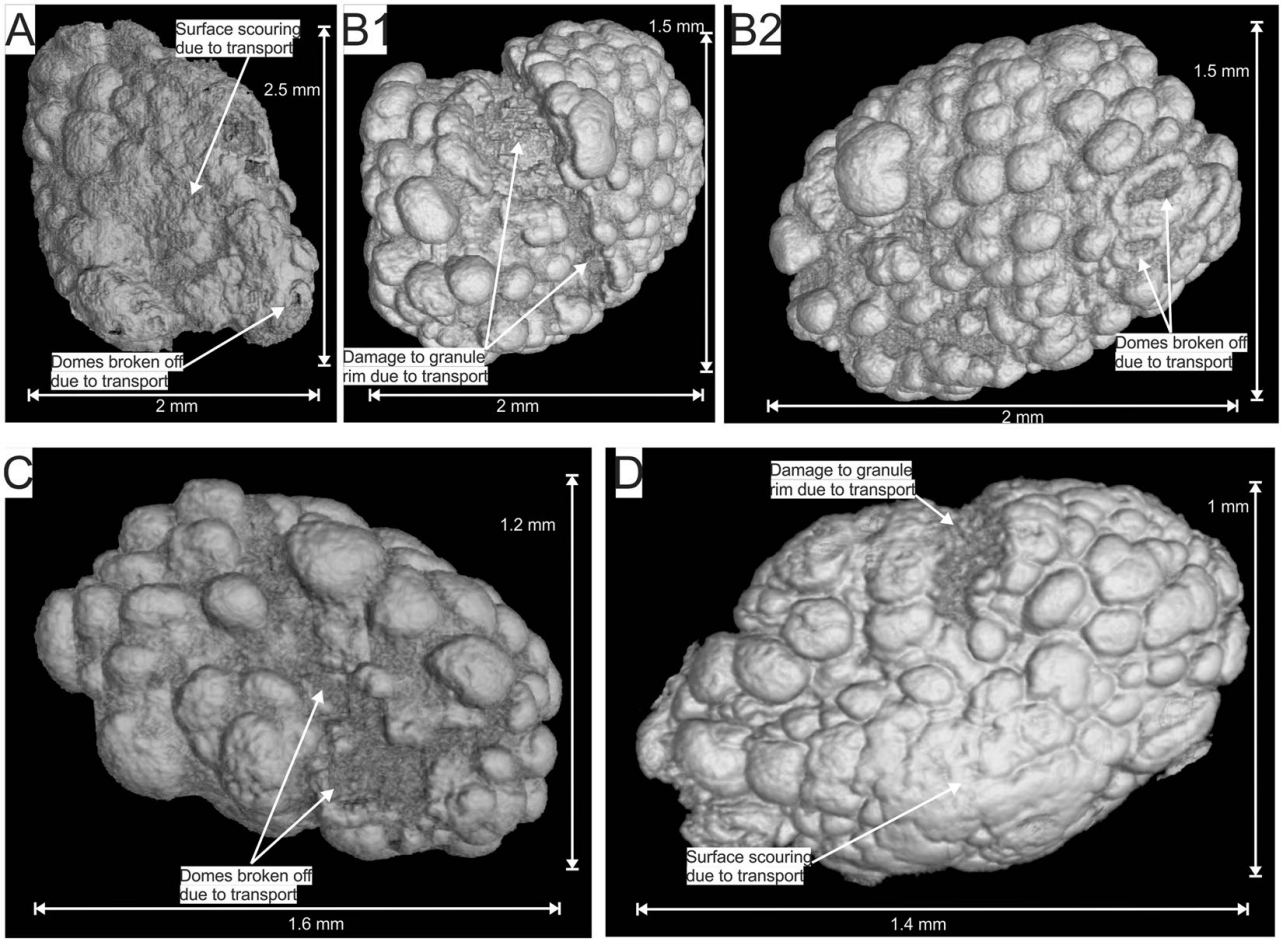
False colour three dimensional reconstructions of µXCT scans of the same four magnetite coated granules granules from Fig. 2 from drill core samples of the Nconga Formation GIF illustrating different alterations to the outer surface of the granules and the domical structures (see text for more detail). See the following supplementary videos for full rotational animations of the extracted granules: 6 for granule A; 8 for granule B; 10 for granule C; 11 for granule D. The images were created using VGStudio Max version 3.2 (https://www.volumegraphics.com/en/products/vgstudio-max.html) and the final figure was compiled using CorelDRAW 2017 (www.coreldraw.com).

The 3D µXCT reconstructions also show modifications to the outer surface of the granules and the domical structures (Fig. 4; Supplementary Videos 6, 8, 10 and 11). These modifications take three main forms, namely: i) scouring and smoothing of the outer coating, expressed as the absence (Fig. 4A) or partial smoothing (Fig. 4D) of the domical surface structures; ii) prominent breaks in or the removal of the outer magnetite coating (Fig. 4B1 and D); and iii) damage to the domical surface structures (Fig. 4A,B2 and C).

**Figure 5:**
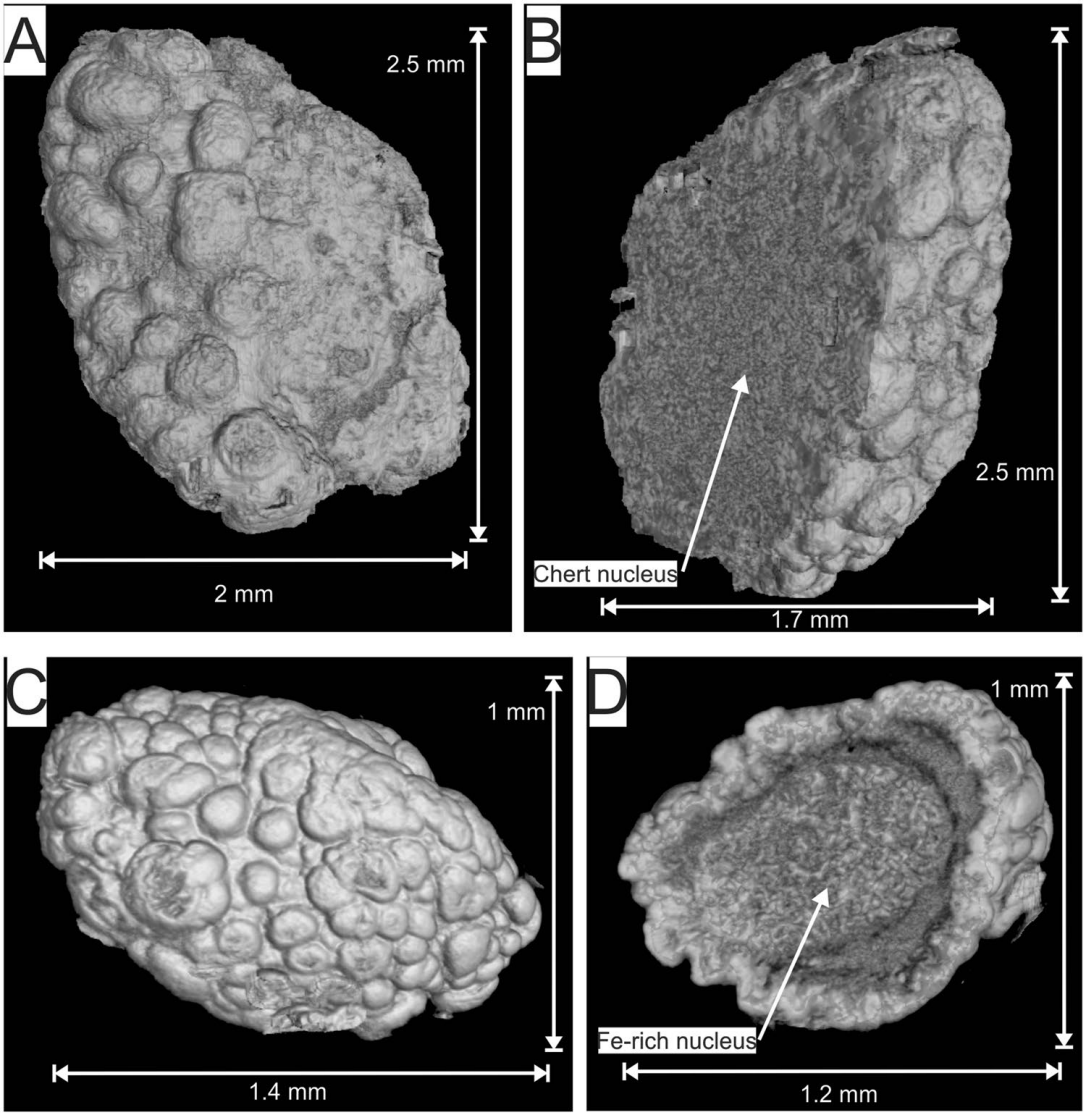
False colour three dimensional reconstruction and cross-sectional cutaway of the µXCT scan of granule A (**A** and **B**) and D (**C** and **D**; Figs. 3–5) from the Nconga Formation GIF illustrating a the more common chert nucleus^25^ (**B**); and the less common magnetite-rich nucleus (**D**), the latter coated by chert which, in turn, is coated by magnetite and calcite domical surface structures. See the following supplementary videos for full rotational and cutaway animations of the extracted granules: 6 for A and B; 12 for C and D. The images were created using VGStudio Max version 3.2 (https://www.volumegraphics.com/en/products/vgstudio-max.html) and the final figure was compiled using CorelDRAW 2017 (www.coreldraw.com).

The majority of the granules are composed of chert and minor calcite cores with rims of interlaminated magnetite and calcite^25^ (Supplementary Fig. 1C and E; Fig. 5A and B; Supplementary Video 6). Few of the granules have a more complex internal structure. The granules contain magnetite-rich nuclei, coated by chert which, in turn, is coated by the same magnetite and calcite domical surface structures as observed in all the other granules (Fig. 5D; Supplementary Video 12).

## Discussion

From the 3D µXCT reconstructions of the individual granules in the Nconga Formation GIF (Fig. 3), it can be seen that the surface structures resemble domical microstromatolites^26^. The overall morphology of the granules can therefore be classified as oncoidal, i.e., stromatolites that occur around grains^27^. In conjunction with the previously published highly negative carbonate δ^13^C_PDB_ values (−15.7 to −11.6 ^0^/_00_), suggestive of organic carbon input, and the heavy magnetite δ^56^Fe values (0.39 to 0.45 ^0^/_00_), indicative of partial oxidation of aqueous Fe^2+^, the observed oncoids were most likely formed by iron-oxidizing bacteria populating the exterior surface of chert granules in a shallow water, occasionally wave-agitated depositional environment^25^. The environmental interpretation is supported by sedimentary structures such as cross bedding and ripples observed in the outcrop in the Kubuta-Mooihoek area of Swaziland^25^. The iron-oxidizing bacteria could have been anoxygenic photoferrotrophs^5,6^, as the granules were formed above wave base and, therefore, in the photic zone. However, chemolithoautotrophs, that function under low concentrations of free oxygen^5^, cannot be eliminated as a possibility due to the strong evidence of an oxygen oasis in the Mozaan depositional basin^21–23^ as well as the possible influence of nitrate as an organic carbon oxidant, as suggested by the lack of Fe-bearing carbonates in the rock^25^.

The granule morphology is also not typical of other GIF occurrences around the world, where the granule rims appear smooth and rounded^28–30^. Microbialite knobby surfaces are known from younger, larger oncoids^31^ (Supplementary Fig. 2), microbial mats^32^ and as bumpy surfaces on columnar stromatolites^33^. More recently formed (Phanerozoic) shallow marine ferromanganese oncoids, which have been linked to microbial mediation in their formation, also show similar-looking external knobby surfaces^34,35^. There are also some depositional setting similarities between these Phanerozoic ferromanganese oncoids and the Nconga GIF granules, sharing a shallow marine setting above or close to wave base^25,34,35^. However, the more recent ferromanganese oncoids are different from the Nconga GIF granules in that the ferromanganese oncoids are different in size, either being orders of magnitude larger^34^ or smaller^35^ than the Nconga GIF granules. Additionally, the ferromanganese oncoids typically have concentric internal structures^34,35^ and can also have high clastic inputs^35^, which are both absent in the Nconga GIF granules^25^.

An alternative interpretation for the morphology of the granules could be that they are hydrothermal siliceous botryoidal sinters, also termed geyserites^36–38^, and therefore potentially do not mark biological structures^38^. There are, however, numerous differences between the Nconga GIF oncoids and botryoidal siliceous sinters that make this interpretation unlikely. Firstly, botryoidal siliceous sinters show a different morphology to that of the Nconga GIF oncoids, where the former comprise agglomerated microspheres, typically forming the whole granule and not only the surface coating^37,38^, and the latter show individual domical microstructures with limited coalescence occurring as a granule coating (Fig. 3). Secondly, the botryoidal siliceous sinters are entirely composed of silica^37,38^, whereas the Ngona GIF granules contain significant magnetite and calcite that mostly occur as the granule coatings. Thirdly, hydrothermal sinters have marked differences in their geochemical composition when compared to the Nconga GIF. For example, hydrothermal sinters tend to have enriched trace metal (e.g. mean Cu contents of ~20–160 ppm) and alkali earth metal contents (mean K_2_O and Na_2_O contents of ~0.10–0.45 and ~0.12–1.14 wt% respectively) with low Fe_2_O_3_ contents^38^ (mean Fe_2_O_3_ contents of ~0.16–6.51 wt%). The Nconga GIF, in contrast, generally have lower trace (e.g. Cu contents of 1.5 and 2.5 ppm) and alkali metal (K_2_O and Na_2_O contents of 0.02–0.03 wt% and below detection limits respectively) contents and higher Fe_2_O_3_ contents^25^ (17.26 and 24.03 wt%).

The 3D µXCT reconstructions of the individual granules also provide unique insight into their sedimentary history. The surface modification of the granule coatings in the form of scouring (Fig. 4A), smoothing (Fig. 4D), rim removal (Fig. 4B1 and D), and microstromatolite damage (Fig. 4B2 and C) illustrate that the granules experienced significant transport after their formation. In addition, the granules occur in upward-fining stacked beds ending in iron-rich mudstone (Supplementary Fig. 1A and E) stratigraphically juxtaposed next to deeper-water iron formations^25^, show multiple orientations (Fig. 2) and are chert matrix-supported (Supplementary Fig. 1A,B and E; Fig. 3). These characteristics all indicate the GIF beds were deposited as small, high-density turbidity flows, likely washed in from an original shallower water setting.

The internal 3D structure of the granules revealed that a minority of granules comprise magnetite-rich nuclei, coated by chert which, in turn, is coated by the same magnetite and calcite domical surface structures observed on all the granules (Fig. 5D). These granules have a more complex, polystadial formational history than the more common granules with chert nuclei (Fig. 5B). A possible interpretation is that a combination of chemical precipitation and sedimentary reworking has formed a composite granule, where an already existing oncoidal granule has been recoated by chert under wave agitation, with another generation of oncoidal laminae growing on the second generation external chert surface. Why only a minority of the granules has this more complex internal structure is not clear. One possibility is that due to density differences between the composite granules (denser due to higher magnetite contents) and the chert nucleus granules, sedimentary sorting mechanisms during transport concentrated the composite granules in a place either removed by erosion at a later stage or not intersected by the drill core.

## Conclusion

This study is the first to document successful 3D µXCT reconstructions of Archean microscale biological and sedimentary structures. In conjunction with the previously published stratigraphy, mineralogy and geochemistry of the Nconga Formation GIF, the 3D morphology of the granules suggest a mixed biological, chemical and sedimentary origin for this unique unit. It is also, at approximately 3 Ga, the oldest documented example of linking iron oxidation and precipitation to the formation of stromatolitic (oncoidal) structures in a demonstrably shallow-water environment. The results broaden our understanding of the environments for biologically mediated iron precipitation in the Archean, which included shallower marine shelf environments where bacterial iron precipitation could take place around granules in a wave-agitated water column. This happened coevally with the more commonly observed deeper water, below-wave base iron precipitation preserved in micritic IF.

## Methods

For this study, three samples, one from outcrop from the Kubuta-Mooihoek area of Swaziland (Supplementary Fig. 1E) and two from an exploration drill core (Figs. 2–5) drilled 20–25 km north northeast of Nongoma in the KwaZulu-Natal Province of South Africa^25^, were selected and cut into blocks for µXCT. The surface sample was cut into an approximately 30 × 30 × 55 mm block, whereas the drill core samples were cut into approximately 7 × 7 × 9 mm blocks. The reason for the latter blocks being smaller was that they could be placed closer to the X-Ray source in order to obtain, through geometric enlargement, higher spatial resolution imaging of individual granules.

µXCT is a well-established analytical and visualization technique that has been applied within the geosciences that enables 3D information to be extracted in a non-destructive manner^39^. X-ray photoelectric absorption and Compton scattering are the most important principles in which X-rays are attenuated by material and are linked to material density in a linear relationship^40^. All the scans were conducted using an industrial Nikon XTH 225 ST µXCT machine located at the South African Nuclear and Energy Corporation (Necsa) at Pelindaba, Northwest Province, South Africa. The Nikon XTH 225 ST µXCT machine was fitted with a Perkin Elmer 1620 amorphous silicon detector as well as a 0.25 mm aluminium filter. The scans were conducted at 100 kV (except for a surface sample that was done at 140 kV) and 100 mA with a scan frame rate of two frames per second. Scan time was 33 minutes and there were 1000 projections per sample. Reconstruction into a 3D virtual sample was performed using Nikon CTPro 3D reconstruction software^41^ while analysis was conducted in VGStudio Max3.2 analytical software^42^. Scanning potential and current parameters for this study were 100 kV and 100 µA respectively, which are based on the contrast observed in the X-ray projection images with a spatial resolution of 6.55 μm.

## Supplementary information

Supplementary information.

Supplementary information 4.

Supplementary information 5.

Supplementary information 6.

Supplementary information 7.

Supplementary information 8.

Supplementary information 9.

Supplementary information 10.

Supplementary information 11.

Supplementary information 12.

Supplementary information 13.

Supplementary information 14.

Supplementary information 15.

## Data Availability

All applicable data is in the manuscript and supplementary files. The original raw 3D µXCT scan data (~50 Gb) can be made available by the corresponding author upon request via a data depository.
