# Supplementary material for: Life on a Mesoarchean marine shelf – insights from the world’s oldest known granular iron formation
Source: Sci Rep. 2020 Jun 29;10:10519. doi: 10.1038/s41598-020-66805-0 (PMC7324406; doi:10.1038/s41598-020-66805-0)
Supplement: Supplementary file 1 — Supplementary information. [file 41598_2020_66805_MOESM1_ESM.docx]

# Life on a Mesoarchean marine shelf – insights from the world’s oldest known granular iron formation

Albertus J.B. Smith^1,2*^, Nicolas J. Beukes^1,2^, Jens Gutzmer^1,2,3,4^, Clark M. Johnson^5^, Andrew D. Czaja^5,6^, Noah Nhleko^1,7^, Frikkie de Beer^8,9^, Jakobus W. Hoffman^8^, Stanley M. Awramik^10^

^1^Paleoproterozoic Mineralization Research Group, Department of Geology, University of Johannesburg, Johannesburg, South Africa

^2^Department of Science and Technology – National Research Foundation Centre of Excellence for Integrated Mineral and Energy Resource Analysis, University of Johannesburg, Johannesburg, South Africa

^3^Helmholtz Zentrum Dresden-Rossendorf, Helmholtz Institute Freiberg for Resource Technology, Freiberg, Germany

^4^Department of Mineralogy, TU Bergakademie Freiberg, Freiberg, Germany

^5^Department of Geoscience, University of Wisconsin, Madison, WI, USA

^6^Department of Geology, University of Cincinnati, Cincinnati, OH, USA

^7^Geological Survey and Mines Department, Mbabane, Swaziland

^8^Radiation Science, South African Nuclear Energy Corporation SOC Ltd. (Necsa), Pelindaba Industrial Site, South Africa

^9^ Senior Research Associate, Department of Anthropology & Development Studies, University of Johannesburg

^10^Department of Earth Sciences, University of California, Santa Barbara, CA, USA

*Corresponding author: A.J.B. Smith; Email: [bertuss@uj.ac.za](mailto:bertuss@uj.ac.za)

## Supplementary information


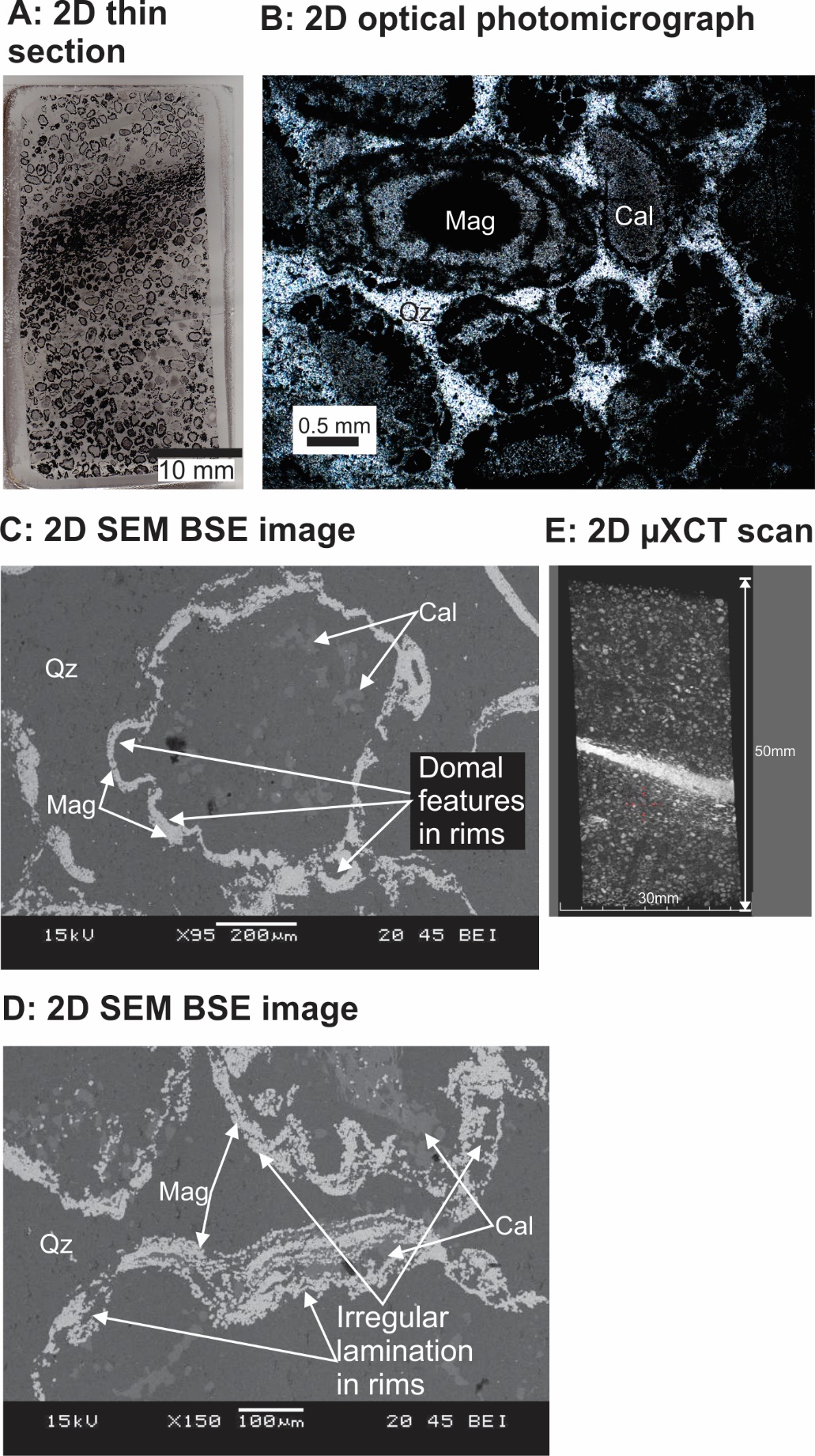


Supplementary Figure 1: A) Scan of a prepared thin section (cross section orientated the right way up) of a drill core sample of the Nconga Formation GIF illustrating an upward fining bed capped by finer granules and some mud, followed by another upward fining bed^1^. B) Plane polarized transmitted light photomicrograph of a drill core sample of the Nconga Formation GIF. Important to note is that the granule cores are dominantly chert (C) and the darker grey and black colours are optical effects of magnetite (black) and calcite (creamy grey) in the granule rims. C and D) Zoomed in SEM BSE images of an individual granule (C) and granule rims (D) in a drill core sample of the Nconga Formation GIF illustrating: chert cores with minor calcite (C); and exterior rims of magnetite with interior rims of calcite or calcite interlayered with magnetite where rims are thicker (D)^1^. E) A single two dimensional slice (internal cross section; sample is the right way up) of an outcrop sample of the Nconga Formation GIF acquired using µXCT, illustrating two upward fining beds, with the lower one capped by iron-rich mudstone (bright band in middle). Cal: calcite; Mag: magnetite; Qz: quartz. See supplementary videos 1 and 2 for two dimensional slices (Video 1) and a three dimensional reconstruction of the sample illustrated in E. The image in (E) was created using VGStudio Max version 3.2 (<https://www.volumegraphics.com/en/products/vgstudio-max.html>) and the final figure was compiled using CorelDRAW 2017 (www.coreldraw.com).


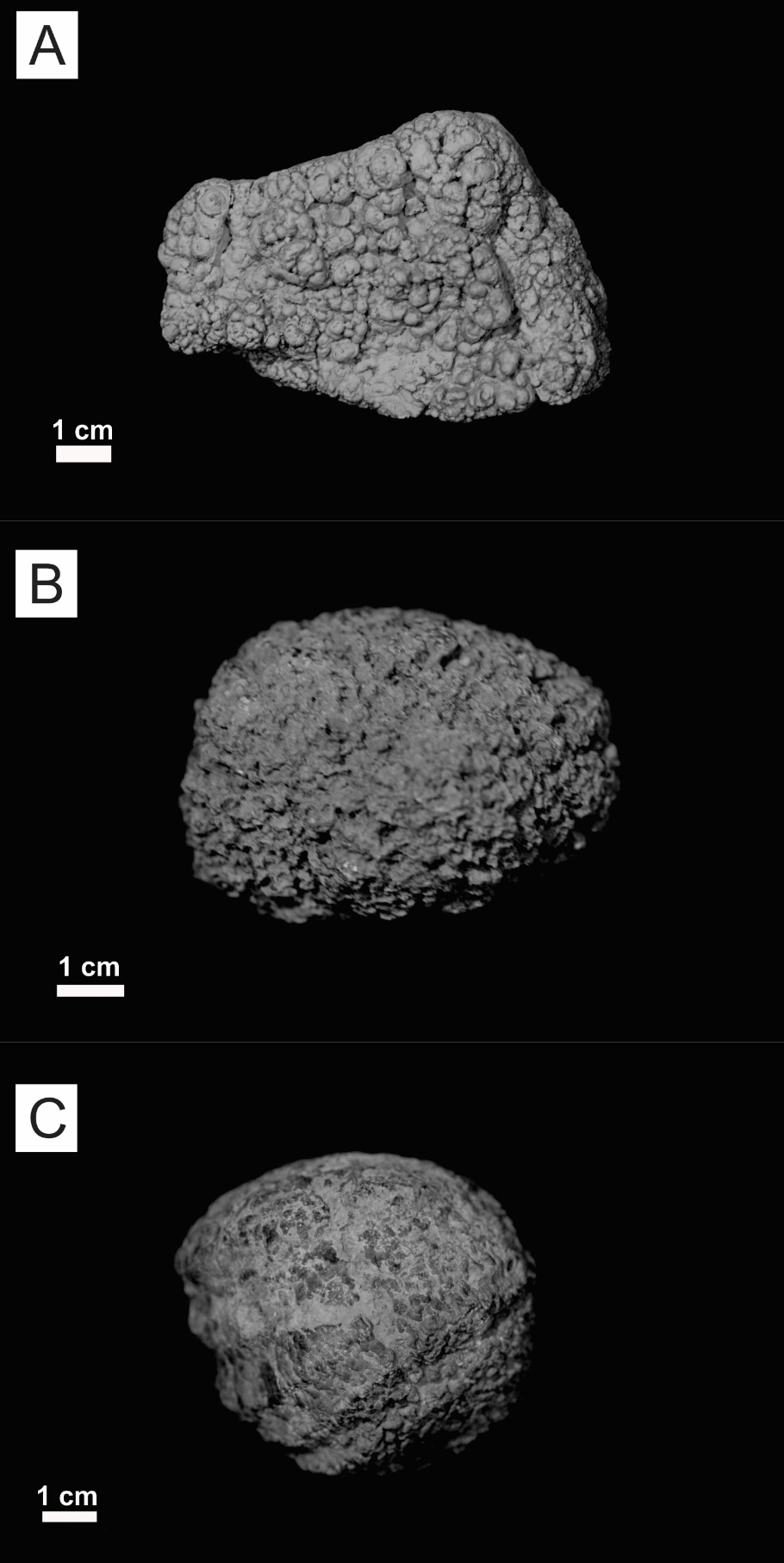


Supplementary Figure 2: Photographs of larger examples of: A) a lacustrine oncoid from the Pleistocene Manix Formation, San Bernardino County, California, USA^4^; B) a lacustrine oncoid from the Cretaceous Lady of Angels Lake, unknown formation, Ascension, Chihuahua, Mexico; and C) a marine *Somphospongia* oncoid, unknown formation, Jackson County, Kansas, USA. The figure was compiled using CorelDRAW 2017 (www.coreldraw.com).

## Supplementary information references

1. Smith, A.J.B. *et al.* Oncoidal granular iron formation in the Mesoarchaean Pongola Supergroup, southern Africa: Textural and geochemical evidence for biological activity during iron deposition. *Geobiology* **15**, 731-749 (2017).
2. Awramik, S.M., Buchheim, H.P., Leggit, L. & Woo, K.S. Oncoids of the Late Pleistocene Manix Formation. *San Bernardino County Museum Ass. Quart.* **44**, 25-31 (2000).

## Supplementary information video captions

Supplementary Video 1: A two dimensional run through animation of the slices acquired using µXCT scanning of one of the outcrop samples from the Nconga Formation GIF. This sample is illustrated in figure 1 F. The frames for the video were created using VGStudio Max version 3.2 (<https://www.volumegraphics.com/en/products/vgstudio-max.html>).

Supplementary Video 2: A rotational and cutaway animation of the three dimensional reconstruction of the µXCT scan of one of the outcrop samples from the Nconga Formation GIF. This sample is illustrated in figure 1 F. The frames for the video were created using VGStudio Max version 3.2 (<https://www.volumegraphics.com/en/products/vgstudio-max.html>).

Supplementary Video 3: A two dimensional run through animation of the slices acquired using µXCT scanning of one of the scanned drill core samples from the Nconga GIF. This sample and granules within it are illustrated in the following figures: 3 A and B; 4 A and B; 5 A, B1 and B2; and 6 A and B. The frames for the video were created using VGStudio Max version 3.2 (<https://www.volumegraphics.com/en/products/vgstudio-max.html>).

Supplementary Video 4: A rotational animation of the three dimensional reconstruction of the µXCT scan of the first scanned drill core samples from the Nconga GIF. This sample and granules within it are illustrated in the following figures: 3 A and B; 4 A and B; 5 A, B1 and B2; and 6 A and B. The frames for the video were created using VGStudio Max version 3.2 (<https://www.volumegraphics.com/en/products/vgstudio-max.html>)..

Supplementary Video 5: A rotational and cutaway animation of the three dimensional reconstruction of the µXCT scan of the first scanned drill core samples from the Nconga GIF illustrating the position and orientation of granule A. The frames for the video were created using VGStudio Max version 3.2 (<https://www.volumegraphics.com/en/products/vgstudio-max.html>).

Supplementary Video 6: A rotational and cutaway animation of the three dimensional reconstruction of the µXCT scan of granule A illustrating the surface morphology of the magnetite coating and an internal nucleus comprising chert. The frames for the video were created using VGStudio Max version 3.2 (<https://www.volumegraphics.com/en/products/vgstudio-max.html>).

Supplementary Video 7: A rotational and cutaway animation of the three dimensional reconstruction of the µXCT scan of the first scanned drill core samples from the Nconga GIF illustrating the position and orientation of granule B. The frames for the video were created using VGStudio Max version 3.2 (<https://www.volumegraphics.com/en/products/vgstudio-max.html>).

Supplementary Video 8: A rotational animation of the three dimensional reconstruction of the µXCT scan of granule B illustrating the surface morphology of the magnetite coating. The frames for the video were created using VGStudio Max version 3.2 (<https://www.volumegraphics.com/en/products/vgstudio-max.html>).

Supplementary Video 9: A rotational and cutaway animation of the three dimensional reconstruction of the µXCT scan of the second scanned drill core samples from the Nconga GIF illustrating the position and orientation of granule C and D. The frames for the video were created using VGStudio Max version 3.2 (<https://www.volumegraphics.com/en/products/vgstudio-max.html>).

Supplementary Video 10: A rotational animation of the three dimensional reconstruction of the µXCT scan of granule C illustrating the surface morphology of the magnetite coating. The frames for the video were created using VGStudio Max version 3.2 (<https://www.volumegraphics.com/en/products/vgstudio-max.html>).

Supplementary Video 11: A rotational animation of the three dimensional reconstruction of the µXCT scan of granule D illustrating the surface morphology of the magnetite coating. The frames for the video were created using VGStudio Max version 3.2 (<https://www.volumegraphics.com/en/products/vgstudio-max.html>).

Supplementary Video 12: A rotational and cutaway animation of the three dimensional reconstruction of the µXCT scan of granule D illustrating the surface morphology of the magnetite coating and an internal polystadial nucleus comprising inner magnetite and outer chert. The frames for the video were created using VGStudio Max version 3.2 (<https://www.volumegraphics.com/en/products/vgstudio-max.html>).
